# Wissenstranslation am Beispiel Bewegungsförderung von älteren Menschen: Wie gelangen wissenschaftliche Erkenntnisse in die kommunale Praxis?

**DOI:** 10.1007/s00103-021-03311-2

**Published:** 2021-04-09

**Authors:** Annalena Bußkamp, Claudia Vonstein, Judith Tillmann, Christin Roßmann, Freia De Bock

**Affiliations:** 1 Referat 2-22 „Zusammenarbeit mit Ländern, Krankenkassen und Verbänden, Gremien; Gesundes Alter; Frauengesundheit; Männergesundheit“, Bundeszentrale für gesundheitliche Aufklärung, Maarweg 149–161, 50825 Köln, Deutschland; 2 Abteilung 2, Bundeszentrale für gesundheitliche Aufklärung (BZgA), Köln, Deutschland

**Keywords:** Evidenzbasierte Praxis, Körperliche Aktivität, Gesundheitsförderung, Public Health, Gesundes Altern, Evidence-based practice, Physical activity, Health promotion, Public health, Healthy aging

## Abstract

**Hintergrund:**

Wissenschaftliche Ergebnisse können eine Wissensquelle für kommunale Akteurinnen und Akteure der Bewegungsförderung sein, finden jedoch aufgrund von vielfältigen Barrieren selten Anwendung. Wissenstranslation kann diesen Prozess vereinfachen, setzt aber das Erfassen der bisher kaum erforschten Bedürfnisse der Akteurinnen und Akteure voraus.

**Ziel der Arbeit:**

Ziel der qualitativen Studie ist es, die Zugangswege der Akteurinnen und Akteure zu Informationen und wissenschaftlichen Erkenntnissen zu erfassen, mögliche Barrieren zu identifizieren sowie die Bedürfnisse der praktisch Anwendenden bezüglich der Darstellung und Aufbereitung herauszustellen.

**Material und Methoden:**

Es wurden leitfadengestützte Interviews mit 12 Kommunal- und Landesakteurinnen und -akteuren der Bewegungsförderung aus Nordrhein-Westfalen, Sachsen-Anhalt und Thüringen geführt. Die Auswahl der Interviewten fand durch Purposive Sampling (gezielte Auswahl der Personen) statt. Die Interviews wurden mittels qualitativer Inhaltsanalyse ausgewertet.

**Ergebnisse:**

Der Nutzen wissenschaftlicher Erkenntnisse wird von den Interviewten betont, jedoch erschweren Ressourcenmangel in Kombination mit Informationsflut, hoher Komplexität und Fachsprache die Anwendung. Es besteht Bedarf an passgenauer Aufbereitung in Form von Zusammenfassungen, Filterfunktionen, Herausarbeiten von praxisrelevanten Elementen und Wegen der Bereitstellung.

**Diskussion:**

Für eine erfolgreiche Wissenstranslation sind die Zusammenarbeit und der interaktive Austausch zwischen Wissenschaft, Politik und Praxis sowie die bedarfsgerechte Aufbereitung von wissenschaftlichen Erkenntnissen zentral. Das Vernetzen sowie Bündeln von Wissen auf einer Plattform sind wichtige Aufgaben für die Zukunft.

**Zusatzmaterial online:**

Zusätzliche Informationen sind in der Online-Version dieses Artikels (10.1007/s00103-021-03311-2) enthalten.

## Abstract (en)

**Background:**

Scientific findings can be an important source of knowledge for public health stakeholders involved in promoting physical activity, but several barriers hinder their use. Knowledge translation can simplify this process, but it requires the understanding of the stakeholder’s needs.

**Objectives:**

This qualitative study aims to describe how public health stakeholders access information and scientific findings, identify possible barriers, and highlight the needs of stakeholders in terms of presentation and processing.

**Materials and methods:**

Semi-structured interviews were conducted with twelve local- and state-level stakeholders from North Rhine-Westphalia, Saxony-Anhalt, and Thuringia working in the area of physical activity promotion. The interviewees were selected through purposive sampling. The interviews were evaluated using qualitative content analysis.

**Results:**

The benefits of scientific findings are emphasized by the interviewees, but a lack of resources in combination with a flood of information, high complexity, and technical jargon complicate their application. There is a need for tailored preparation in the form of summaries, filter functions, elaboration of practice-relevant elements, and ways of provision.

**Conclusions:**

To achieve successful knowledge translation, collaboration and interactive exchange between researchers, policymakers, and practice as well as a demand-oriented processing of scientific findings are central. Networking and bundling of knowledge on a platform are important tasks for the future.

## Einleitung und Hintergrund

Wissenschaftliche Erkenntnisse (WE) können einen wichtigen Beitrag zur evidenzinformierten Entscheidungsfindung^1^ [1] von kommunalen Akteurinnen und Akteuren (AK) der Bewegungsförderung mit Entscheidungskompetenzen leisten [2], finden jedoch zum Teil nur in geringem Maße Beachtung [3]. Zu den Gründen, die bisher insbesondere für Entscheidungstragende im internationalen Raum dokumentiert wurden, zählen vielfältige Barrieren, u. a. Ressourcenknappheit, fehlender Zugang zu den WE und deren praxisferne bzw. unzureichend an den Bedürfnissen der Nutzenden orientierte Aufbereitung in Bezug auf Länge, Fachjargon und Inhalte [2, 4–8]. Bisherige Untersuchungen zeigen, dass Entscheidungen in der Praxis insbesondere auf persönlichen Erfahrungen, Standardarbeitsweisen und anekdotischen Erzählungen basieren [9–11], die teilweise im Gegensatz zu wissenschaftlichen Prinzipien [11, 12] und einer evidenzinformierten Entscheidungsfindung [1] stehen. Die Berücksichtigung von WE in der Praxis hat den Vorteil, dass mit einer höheren Wahrscheinlichkeit wirksame Public-Health-Maßnahmen durchgeführt, Opportunitätskosten gesenkt und folglich öffentliche und private Ressourcen effizienter genutzt werden können [9]. Wissenstranslation^2^ kann hier zu einem besseren Verständnis, einer breiteren Nutzung und Akzeptanz der WE in der Praxis, zur Verringerung der Lücke zwischen Wissen aus der Forschung und Umsetzung in der Praxis und somit zur Verbesserung der Public-Health-Maßnahmen führen [13, 14]. Laut Straus et al. [3] meint Wissenstranslation die Anwendung von Wissen in der Praxis und bei der Entscheidungsfindung durch AK. Die Canadian Institutes of Health Research führen diese Definition weiter und bezeichnen sie als jeden dynamischen, iterativen Prozess, der die Synthese, die Disseminierung, den Austausch und die ethisch angemessene Anwendung von Wissen beinhaltet, um die Gesundheit der Bevölkerung zu verbessern, effektivere Gesundheitsservices und -produkte bereitzustellen und das Gesundheitssystem zu stärken [15]. Der Prozess ist dabei in ein komplexes System aus Interaktionen zwischen Wissensnutzenden und Forschenden eingebettet, die in ihrem Ausmaß an Engagement, Intensität und Komplexität in Abhängigkeit der Bedürfnisse, Ergebnisse und Art der Forschung variieren [14–16].

Insbesondere im internationalen Kontext wird über Wissenstranslation und die Aufbereitung von WE publiziert. Diese ersten Erkenntnisse lassen sich jedoch nicht uneingeschränkt auf Deutschland und das Feld der Bewegungsförderung übertragen. Der Grund hierfür sind unter anderem kontextuelle Rahmenbedingungen des Gesundheitssystems, föderale Strukturen, unterschiedliche AK und Schwerpunkte der Public-Health-Bemühungen. Die vorliegende Studie untersucht daher unserer Kenntnis nach zum ersten Mal in Deutschland, welche konkreten Bedürfnisse zur Aufbereitung und Bereitstellung von WE kommunale AK der Bewegungsförderung haben und wie die Nutzung von WE in der Praxis durch verbesserte Translation erleichtert werden kann. Die AK sind dabei Personen aus der Praxis, die für die Planung, Umsetzung und Strukturentwicklung von Maßnahmen der kommunalen Bewegungsförderung von älteren Menschen in Lebenswelten zuständig sind und zum Teil (politische) Entscheidungskompetenzen besitzen. Im Rahmen des Programms „Älter werden in Balance“ der Bundeszentrale für gesundheitliche Aufklärung (BZgA), welches sich mit der beschriebenen Akteursgruppe beschäftigt (siehe auch https://www.aelter-werden-in-balance.de), sollen hierzu folgende wissenschaftliche Fragestellungen beantwortet werden:

- Wie beschaffen kommunale AK Informationen und Daten zu möglichen Maßnahmen der Bewegungsförderung von älteren Menschen?
- Welchen Nutzen sehen diese in WE?
- Welche Barrieren bestehen bei der Beschaffung und Nutzung von WE?
- In welcher Form müssen WE aufbereitet sein, um praxisrelevant und verständlich für AK der Bewegungsförderung zu sein?

Die vorliegende Studie orientiert sich dabei an einem konzeptionellen Rahmen von Ellen et al. [17], der 7 Elemente zur Schließung der beschriebenen Lücke enthält:

1. Berücksichtigung des lokalen Kontexts,
2. Aufbau von Beziehungen zwischen Forschenden und AK,
3. Erstellung von relevantem und aktuellem Wissen,
4. „Push“-Aktivitäten^3^ von Forschenden,
5. Erleichterung von „Pull“-Bemühungen,
6. „Pull“-Aktivitäten der AK,
7. Evaluation der genannten Aktivitäten.

Die Arbeit untersucht insbesondere das dritte Element.

## Methodisches Vorgehen

Zur Erforschung des Gebietes wird ausgehend von der Consolidated-Criteria-for-Reporting-Qualitative-Research-Checkliste [18] auf ein qualitatives Forschungskonzept zurückgegriffen. Dieses enthält als Instrument der Datenerhebung leitfadengestützte Experteninterviews mit offenen Leitfragen und Reflexionsbögen und stützt sich methodologisch auf die qualitative Inhaltanalyse von diesen. Vor den Interviews wurden Kurzfragebögen mit soziodemografischen Daten ausgefüllt, um die Heterogenität der AK abzubilden. Die Expertinnen und Experten sind Mitarbeitende von Kommunalverwaltungen (AK erster Ordnung^4^ [19]) sowie von nichtstaatlichen Landesverbänden (AK zweiter Ordnung) aus Nordrhein-Westfalen, Sachsen-Anhalt und Thüringen. Sie alle weisen Expertise im Bereich der Bewegungsförderung von älteren Menschen auf.

Die AK auf Landesebene sind stark mit kommunalen AK vernetzt und verfügen über ein großes Wissen bzgl. der vorhandenen Strukturen und Probleme. Die kommunalen AK (Verwaltungsmitarbeitende) kennen die präzisen Entscheidungsabläufe in ihren Kommunen, weshalb sie Auskunft über das eigene Handlungsfeld sowie die Bedürfnisse aus der Praxis geben können. Die für diese Studie ausgewählten AK arbeiten in Gesundheitsämtern, Bauämtern, dem Bürgerservice, Fachbereichen für Soziales, Arbeit und Senioren und Beratungsstellen für Sport- und Bewegungsbetreuung.

**Figure 1:**
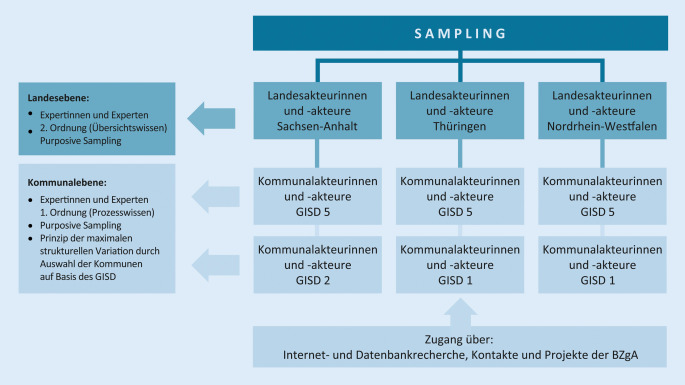

Basierend auf vorhandenen WE [2, 7, 8, 13, 20] und eigenen Erfahrungen im Rahmen des Programms „Älter werden in Balance“ wurde ein semistrukturierter Interviewleitfaden erstellt. Die Expertinnen und Experten auf Landesebene wurden über bestehende Kontakte und Kooperationen gezielt ausgewählt (Purposive Sampling). Expertinnen und Experten auf Kommunalebene wurden auf Basis der größtmöglichen Variation der Fälle und Kontexte innerhalb des sozioökonomischen Deprivationsindexes für Deutschland des Robert Koch-Instituts auf Bundeslandebene aus dem Jahr 2014 [21] ebenfalls gezielt ausgewählt (Purposive Sampling). Abb. 1 fasst das Vorgehen zusammen. Die Kontaktaufnahme bei 85 Personen erfolgte telefonisch oder per E‑Mail (Rückmeldung von 35 Personen), dabei wurden Ziele und Hintergründe der Interviews vorgestellt und datenschutzkonforme Einwilligungserklärungen per E‑Mail versendet. Bei schriftlicher Einwilligung zur Teilnahme wurde ein Interviewtermin vereinbart. Als Gründe für die Nichtteilnahme wurden u. a. fehlende zeitliche Ressourcen und Kompetenzen von den AK angegeben.

Insgesamt wurden zwischen Juni 2019 und August 2020 10 telefonische und persönliche Interviews im Arbeitsumfeld von 12 Expertinnen und Experten bis zur thematischen Sättigung von der Erstautorin durchgeführt. Bei den Interviews handelt es sich zumeist um Einzelinterviews, lediglich ein Interview wurde als Gruppeninterview geführt. Die Dauer betrug zwischen 15 min und 54 min. Die Interviews wurden tontechnisch aufgezeichnet, transkribiert und mittels qualitativer Inhaltsanalyse nach Gläser und Laudel [22] im Vieraugenprinzip mithilfe von Microsoft Excel (Microsoft Corporation, Redmond, WA, USA) codiert und ausgewertet (siehe Onlinematerial Tabelle Z1). Die qualitative Inhaltsanalyse enthielt dabei 5 zentrale Schritte: theoretische Vorüberlegungen, Vorbereitung der Extraktion, Extraktion, Aufbereitung und Auswertung (siehe Beschreibung der Datenanalyse im Onlinematerial).

## Ergebnisse

Im Folgenden wird ein Teil der zentralen Ergebnisse der Studie anhand der Kategorien Informationsbeschaffung und WE (Tab. 1) und den im Onlinematerial Tabelle Z1 genannten Dimensionen vorgestellt. Tab. 1 und Tabelle Z1 fassen die verschiedenen Kategorien und Subkategorien mit beispielhaften Zitaten zusammen. Die soziodemografischen Merkmale der Teilnehmenden können Tab. 2 entnommen werden.

1. Kategorie Informationsbeschaffung

**Table 1:** 

Kategorie	Beschreibung der Kategorie	Subkategorie	Beschreibung der Subkategorie	Zitat
Informationsbeschaffung	Prozess der Gewinnung von Informationen, d. h. von kontextabhängigen Daten, der die folgenden Schritte enthält: Erschließen von Informationsquellen Sammeln von Informationen Aufbereiten von Informationen Bewerten von Informationen	Rahmenbedingungen	Faktoren, die die Informationsbeschaffung der kommunalen Akteurinnen und Akteure positiv und negativ beeinflussen	„Ich … [realisiere zwar], dass Informationen da sind …, aber ich habe gar keine Zeit, mich da innerlich mit zu befassen, und habe … 2 Schrankbereiche, wo ich immer alles hinlege und sage später mal. Und das wird immer später“* (Person 1)* „Jemand, der in der Stadtverwaltung arbeitet, kann mit … [wissenschaftlichen Erkenntnissen], was ganz anderes anfangen, als jemand, der in einer Senioreneinrichtung sich um Nachmittage kümmert“* (Person 3)*
		Zugang	Zugangswege und Wissensträger in Kommunen, über welche sich kommunale Akteurinnen und Akteure Informationen zur kommunalen Bewegungsförderung beschaffen	„Wenn es mal um konkretere Sachen geht, fragt man auch mal Mister Google“ (Person 8) „Grundsätzlich [gibt es] verschiedene Möglichkeiten, über die zuständigen Ministerien und Städte- und Gemeindebund an … [Informationen] heranzukommen“ (Person 5)
Wissenschaftliche Erkenntnisse	Bestverfügbare Ergebnisse aus wissenschaftlichen Studien, von Fachinstitutionen oder Fachleuten, die zur Beantwortung einer wissenschaftlichen Fragestellung mithilfe von qualitativen und/oder quantitativen Methoden erhoben wurden	Nutzen	Vorteil, der durch die Anwendung von wissenschaftlichen Erkenntnissen im Berufsalltag der kommunalen Akteurinnen und Akteure entsteht	„… zumal dann auch die politischen Gremien danach fragen, warum wir was machen. Wenn wir sagen, wir haben hier eine Studie von X, Y, aufgrund dessen bauen wir das auf, dann ist das schon etwas ganz anderes, als wenn wir sagen, ja wir haben uns mal etwas überlegt. Also daher sind solche Studien für uns schon Argumentationshilfe und auch Umsetzungshilfe“* (Person 1)* „Mit der wissenschaftlichen Grundlage … kann man … Begründungen liefern für die weitere Arbeit … Sonst heißt es …: Ihr schwimmt wohl im eigenen Saft. Habt ihr euch das selber ausgedacht? Nein, man muss sportliche Entwicklungsmaßnahmen in einer Kommune … immer auf so einer Basis … relativ neuer wissenschaftlicher Erkenntnisse [machen]“* (Person 5)*
		Einbezug in Entscheidungen	Beachtung von wissenschaftlichen Erkenntnissen bei Entscheidungen über verschiedene Handlungsalternativen der kommunalen Bewegungsförderung	„Das kommt auf die [Akteurinnen und] Akteure in den Verwaltungsstrukturen an. Also ich habe gute Erfahrungen bei manchen Gesundheitsämtern“ (Person 3) „Wenn es darum geht, eine Vorlage auch für den Stadtrat … vorzubereiten, müssen die [Verwaltungsmitarbeitenden] ja wissen, wovon sie reden, damit das im Stadtrat dann nicht einfach plattgemacht wird“ (Person 12)
		Barrieren	Faktoren, die die Nutzung und Anwendung von wissenschaftlichen Erkenntnissen in der kommunalen Bewegungsförderung behindern	„Wenn das zu intensiv beschrieben wird … mit so vielen Fachbegriffen, wo ich fast einen Duden daneben liegen habe, um zu verstehen, was derjenige damit meint. Das hindert mich dann schon so eine Studie von A bis Z zu lesen“ *(Person 1)* „Ich muss Ihnen ganz ehrlich sagen, ich habe bisher noch … wirklich keine Barriere erlebt“* (Person 2)*
		Strukturelle Aufbereitung	Für kommunale Akteurinnen und Akteure ideale Gliederung und Gestaltung der wissenschaftlichen Erkenntnisse	„Es muss halt wirklich kurz und knapp [sein], weil alles andere, das merke ich halt selber in meinem Alltag, lese ich dann selten“ (Person 10) „Zwischen 20 und 30 Seiten lese ich dann schon mal, wenn der Inhalt umfassend ist, speziell auch … auf die Region …, zum Beispiel ländliche Region, zugeschnitten ist“ (Person 5)
		Inhaltliche Aufbereitung	Für kommunale Akteurinnen und Akteure ideale inhaltliche Darstellung der wissenschaftlichen Erkenntnisse	„… dann einfach [schreiben] …, was herausgekommen ist … Weil meistens gibt es ja ein Ergebnis, das lässt sich … in einem Satz zusammenfassen, auch wenn man das nicht hören möchte“* (Person 10)* „Es gibt Tausende gute Beispiele und ich weiß aber auch, dass viele Akteure sofort das Gefühl haben, okay, die und die Ausgangslage ist nicht gleich, das heißt, ich kann damit gar nichts anfangen“* (Person 3)*
		Bereitstellung	Möglichkeiten der Zurverfügungstellung von wissenschaftlichen Erkenntnissen für kommunale Akteurinnen und Akteure	„Am liebsten würde ich mir jemanden wünschen, der an einem Telefon sitzt und der von den Kommunen angerufen werden kann, wo derjenige sagen kann, also spezifisch für meine Kommune bräuchte ich das, das und das“ (Person 3) „Newsletter ist schon okay. Das machen ja inzwischen viele, da haben wir auch Zugang zu und das ist was, da kann man mal schnell … überfliegen. Ist was Interessantes für mich dabei, dann kann ich es mir rausziehen“ (Person 8)

**Table 2:** 

Variable	Anzahl Teilnehmende *n* (%)	Mittelwert $\bar{x}$ mit Standardabweichung (SD)
*Geschlecht*		–
Männlich	5 (41,7)	
Weiblich	7 (58,3)	
*Alter*		46 (± 12,22) ^a^
26–35 Jahre	3 (25,0)	
36–45 Jahre	1 (8,3)	
46–55 Jahre	4 (33,3)	
56–65 Jahre	3 (25,0)	
Keine Angabe	1 (8,3)	
*Berufserfahrung*		20,77 (± 13,42) ^a^
0–10 Jahre	4 (33,3)	
11–20 Jahre	1 (8,3)	
21–30 Jahre	3 (25,0)	
31–40 Jahre	2 (16,7)	
41–50 Jahre	1 (8,3)	
Keine Angabe	1 (8,3)	
*Setting*		–
Land	6 (50,0)	
Kommune	6 (50,0)	
*Stadt- und Gemeindetyp (nach Bundesamt für Bauwesen und Raumordnung)*		–
Großstadt	3 (50,0)	
Mittelstadt	2 (33,3)	
Kleinstadt	1 (16,7)	
Landgemeinde	0	
*Bildungsniveau (nach International Standard Classification of Education (ISCED))*		–
ISCED 4	1 (8,3)	
ISCED 6	2 (16,7)	
ISCED 7	7 (58,3)	
ISCED 8	2 (16,7)	
*Ausbildungsfeld*		–
Sport‑, Gesundheitswissenschaften	5 (41,7)	
Verwaltungswissenschaften	2 (16,7)	
Ingenieurswissenschaften	1 (8,3)	
Sozialwissenschaften	2 (16,7)	
Naturwissenschaften	1 (8,3)	
Sprach‑, Kulturwissenschaften	1 (8,3)	
*Berufstätigkeit*		–
Bereichsleitung	4 (33,3)	
Referentin/Referent	4 (33,3)	
Sachbearbeitung	2 (16,7)	
Sonstiges	2 (16,7)	

^a^fehlende Angabe (*n* = 1)

Um Bewegungsförderungsmaßnahmen in der Kommune etablieren oder weiterentwickeln zu können, müssen kommunale AK Informationen und Daten beschaffen und nutzen. Dabei kommen laut der interviewten AK 5 Zugangswege infrage:

- *Personen* im direkten Umfeld (z. B. Kollegen),
- *Netzwerke* (z. B. Städte- und Gemeindebünde),
- *Internet*,
- *Newsletter* und
- *Fachzeitschriften*.

Die Zugangswege und Rahmenbedingungen sind dabei stark von den jeweiligen AK, ihren Vorkenntnissen, Erfahrungen und Tätigkeitsbereichen abhängig und folglich aufgrund der *Akteursvielfalt *im Bereich der Bewegungsförderung heterogen. Problematisch ist die fehlende Zeit zum Lesen und Verstehen von Informationen und Daten. So werden Materialien durch einen Mangel an *Ressourcen* häufig nicht gelesen. Die AK bemängeln die Masse an Input, die zu einem *Informationsüberfluss* führen kann.

2. Kategorie Wissenschaftliche Erkenntnisse

Die interviewten AK sehen einen Nutzen in WE und verstehen sie insbesondere als *Umsetzungs- und Argumentationshilfe* gegenüber Politik und Einwohnenden. Die WE werden in Entscheidungen einbezogen, die Relevanz schwankt je nach AK und Aufgabenspektrum. Um *politischen Rückhalt* zu erhalten, ist eine wissenschaftliche Fundierung zumeist unumgänglich, jedoch sind die vorhandene* Komplexität* und ein zu großer Umfang in Kombination mit knappen *Ressourcen *oftmals abschreckende Barrieren. *Statistik* und *Fachsprache *sind zum Teil schwer verständlich und WE für die AK schwer zugänglich. Lediglich einer der befragten AK verneint das Vorhandensein von Barrieren.

Zur Beseitigung dieser Barrieren wünschen sich die AK im Sinne der strukturellen Aufbereitung *Suchmasken* und Filter zur Durchsicht der Literatur sowie kurze *Zusammenfassungen* in allgemeinverständlicher Sprache. In Bezug auf die *Länge* sind sich die AK uneinig, sodass eine Spanne zwischen einer und 30 Seiten genannt wird. Die Mehrheit plädiert jedoch für ein möglichst kurzes Format. Die AK merken zur inhaltlichen Aufbereitung an, dass WE Informationen zur *Übertragbarkeit *in den eigenen Kontext, zu *förderlichen und hinderlichen Faktoren, Setting und Rahmenbedingungen* enthalten sollten. Sie sind sich einig, dass Angaben zu *Kosten *(z. B. Kostenarten) für eine bessere Maßnahmenplanung gemacht werden müssten. Eine Aufführung von *Beispielen* sehen alle AK als hilfreich an, währenddessen in Bezug auf die Auflistung von *statistischen Werten* Uneinigkeit besteht. Das *Ergebnis *der WE sollte abschließend kurz und prägnant mit *Handlungsempfehlungen* dargestellt werden.

Ein Großteil der AK bevorzugt die Bereitstellung von WE per *Newsletter*, da nach Inhalten selektiert werden kann und nicht aktiv nach Literatur gesucht werden muss. Lediglich ein Akteur präferiert eine Bereitstellung per* Fachzeitschrift*. Weiterhin sind Transferworkshops auf *Veranstaltungen* und telefonische sowie persönliche *Beratungen* vorstellbar, sodass Fragen direkt beantwortet und Probleme behoben werden können.

## Diskussion

Diese qualitative Studie gibt erstmalig im Bereich der kommunalen Bewegungsförderung in Deutschland Hinweise auf die Komplexität der Zugangswege zu und Aufbereitung von WE. Die Interviewten sehen den Nutzen der Wissenschaft; jedoch bestehen vielseitige Barrieren, die auch in nationalen und internationalen Forschungsarbeiten über verschiedene Zielgruppen hinweg bestätigt wurden [2, 5, 6, 23–29]. Sowohl in dieser als auch in weiteren Untersuchungen konnte festgestellt werden, dass Zusammenfassungen mit entscheidungsrelevanten Angaben in allgemeinverständlicher Sprache signifikant für die Praxis sind und zu einer häufigeren Nutzung führen [2, 4–6, 13, 17, 23, 24, 30, 31]. Die scheinbar widersprüchliche Vorliebe für Kürze und gleichzeitig Detailliertheit wurde auch von anderen Autorinnen und Autoren [6, 31] berichtet. Weitere Studien bestärken zum Teil die von den AK bevorzugten Kanäle für den Erhalt von WE [4, 6, 13, 29]. Diese und andere [4, 9, 29, 32] zeigen jedoch auch, dass Onlinezugänge, Webseiten und soziale Medien wirksamere Strategien für die Verbreitung von WE sind. Die bevorzugten Zugangswege sollten daher spezifisch bei verschiedenen Zielgruppen und Themen erneut abgefragt werden.

Partizipative Wissenstranslation wurde im Rahmen dieser Studie nicht untersucht. In der Literatur gelten als Erfolgsfaktoren für die Nutzung und Akzeptanz von WE ein rechtzeitiger Zugang zu qualitativ hochwertigen und relevanten Forschungsergebnissen, die Zusammenarbeit von Forschenden mit Entscheidungstragenden und der Aufbau von Partnerschaften zwischen Forschenden und praktisch Anwendenden [23, 26, 33–36]. Eine mögliche Schlussfolgerung daraus wäre, Wissenstranslation als zyklisch und nicht unidirektional anzusehen. Dies bedeutet, Wissen nicht nur aus der Forschung in die Praxis zu bringen und von Beginn der Forschungsbemühungen zu berücksichtigen, sondern auch aus der Praxis in die Forschung zu geben [37], damit Bedarfe mitgeteilt und Erfahrungen einfließen können. Insbesondere partizipatorische, interaktive Ansätze, die die AK aktiv in Forschungsprojekte einbeziehen, beispielsweise in Form von What-Works-Papieren aus Kanada [38] oder den Niederlanden [39], haben sich im internationalen Raum im Vergleich zu unidirektionalen Modellen als vielversprechend herausgestellt [9, 40]. Hierbei sollten mögliche Limitationen wie die Kompetenzen, Vorerfahrungen und zeitlichen Ressourcen der AK beachtet und untersucht werden.

Bundesaufgabe kann in einem ersten Schritt daher die Vernetzung von AK der Bewegungsförderung aus Praxis, Politik und Wissenschaft durch bspw. niedrigschwellige Veranstaltungen sein. Persönliche Begegnungen haben sich in der Vergangenheit dabei als effizientester Weg herausgestellt [34, 36]. Des Weiteren bietet sich die Bereitstellung einer einheitlichen, leicht zugänglichen und in Partizipation mit den AK erarbeiteten digitalen Plattform an, die ein standardisiertes Vorgehen der kommunalen Bewegungsförderung trotz Varianz in den Strukturen zulässt, für die Praxis relevante, aktuelle und bedarfsgerecht aufbereitete WE bündelt, ggfs. per Newsletter zur Verfügung stellt und so die „Pull“-Bemühungen der AK fördert. Die Plattform kann zugleich für Feedback und die Entwicklung von praxisbasierten, wissenschaftlichen Fragestellungen in einem bedürfnisgerechten Format (ähnlich What Works) genutzt werden. Eine derartige Plattform (Impulsgeber Bewegungsförderung) wird derzeit u. a. aufbauend auf diesen Ergebnissen im Rahmen einer entsprechenden Implementierungsstrategie von der BZgA entwickelt. Im internationalen Raum haben sich ähnliche Plattformen als hilfreiche Instrumente für unterschiedliche Nutzende herausgestellt [36, 41, 42], die Untersuchung der Effektivität erweist sich jedoch als Herausforderung [36]. Um die Durchdringung der Maßnahmen zu erhöhen, könnte dieses Angebot durch analoge Beratungsleistungen ergänzt werden. Hier könnten beispielsweise, ähnlich zum Konzept des Knowledge Broker [43], Personen oder Organisationen als Wissensvermittler eingesetzt werden. Alternativ ist auch das Angebot von Kompetenzschulungen und Trainings für praktisch Anwendende und Forschende vorstellbar. Integrierte Interventionen aus Kompetenzentwicklung und dem Zugang zu Wissensvermittlern, Ressourcen und Tools haben sich dabei in bisherigen Forschungsarbeiten als vielversprechende Strategie herausgestellt [35].

## Limitationen

Diese Studie spiegelt die vielfältigen Bedürfnisse von interdisziplinär ausgebildeten AK der Bewegungsförderung auf Kommunal- und Landesebene wider. Diese sind jedoch möglicherweise nicht auf andere Themenbereiche außerhalb der kommunalen Bewegungsförderung übertragbar und für AK von Kommunen mit geringen Einwohnendenzahlen (Landgemeinden, Tab. 2) anwendbar. Die AK waren sich bewusst, dass die Interviewerin für eine Bundesbehörde arbeitet, was das Antwortverhalten beeinflusst haben und zu Verzerrungen geführt haben könnte.

## Fazit

Zum ersten Mal wurden in einer qualitativen Studie AK der Bewegungsförderung von älteren Menschen in Deutschland zu den Zugangswegen, der Aufbereitung und Bereitstellung von WE interviewt. Die Translation von WE ist für AK zwar äußerst relevant, jedoch mangelt es an praxisgerechten Aufbereitungen und Zugängen. Damit künftig WE häufiger in der Praxis Anwendung finden und evidenzinformierte Entscheidungen getroffen werden, sollten WE bedarfs- und bedürfnisgerecht aufbereitet und verbreitet werden.

## Supplementary Information



## Footnotes

1 Evidenzinformierte Entscheidungsfindung meint den Prozess der Gewinnung und Verbreitung von bestverfügbaren WE und die Nutzung dieser zur Information und Verbesserung der Public-Health-Bemühungen.

2 Aktivitäten, die darauf abzielen, die Nutzung von WE in die Praxis zu steigern, wurden von anderen je nach Disziplin und Wissenschaftsfeld durch vielfältige Begriffe (z. B. Wissenstransfer, Forschungsimplementierung) beschrieben [14, 16, 37]. Für die Zwecke dieser Studie wird der Begriff „Wissenstranslation“ verwendet und so verstanden, dass er die oben genannten Begriffe umfasst. „Wissen“ inkludiert hier kontextabhängige Informationen und wissenschaftliche Forschung, während der Begriff „Translation“ sich auf den Prozess der Übertragung des Wissens bezieht.

3 „Push“-Aktivitäten sind Bemühungen von Forschungsorganisationen zur Verbreitung von WE, während „Pull“-Aktivitäten Maßnahmen von AK für den Zugang zu und die Nutzung von WE sind.

4 Der wissenssoziologische Expertenbegriff ermöglicht unter Bezugnahme des methodisch-relationalen Ansatzes eine Differenzierung in Expertinnen und Experten erster und zweiter Ordnung. Experten und Expertinnen erster Ordnung sind diejenigen, die insbesondere praxeologisches Betriebswissen, also Prozesswissen, besitzen. Expertinnen und Experten zweiter Ordnung sind solche, die abstrakt-reflexives Kontextwissen, ergo Überblickswissen, aufweisen.
