# Supplementary material for: Promotion of physical activity among the elderly as an example of knowledge translation: How do scientific findings enter community practice?
Source: Bundesgesundheitsblatt Gesundheitsforschung Gesundheitsschutz. 2021 Apr 9;64(5):560–7. [Article in German] doi: 10.1007/s00103-021-03311-2 (PMC8087559; doi:10.1007/s00103-021-03311-2)
Supplement: Supplementary file 1 [file 103_2021_3311_MOESM1_ESM.pdf]

Bundesgesundheitsblatt 5/2021

Elektronisches Zusatzmaterial zum Beitrag:

Wissenstranslation am Beispiel Bewegungsförderung von älteren Menschen: Wie gelangen wissenschaftliche Erkenntnisse in die kommunale Praxis?

Annalena Bußkamp[1], Claudia Vonstein[1], Judith Tillmann[1], Christin Roßmann[1], Freia De Bock[2]

[1] Bundeszentrale für gesundheitliche Aufklärung (BZgA), Referat 2-22, Köln, Deutschland
[2] Bundeszentrale für gesundheitliche Aufklärung (BZgA), Abteilung 2, Köln, Deutschland

Korrespondierende Autorin:

Annalena Bußkamp
Referat 2-22 „Zusammenarbeit mit Ländern, Krankenkassen und Verbänden, Gremien; Gesundes Alter; Frauengesundheit; Männergesundheit"
Bundeszentrale für gesundheitliche Aufklärung
Maarweg 149-161
50825 Köln
Deutschland
annalena.busskamp@bzga.de

Inhalt:
-Tabelle Z1: Auszug aus dem Kodierbaum
-Beschreibung der Datenanalyse

Tabelle Z1: Auszug aus Kodierbaum

Im Rahmen der Auswertung der Interviews wurde auf Basis von theoretischen Vorüberlegungen ein Kategoriensystem entworfen. Ein Auszug aus diesem System mit den für den Artikel relevanten Kategorien, Subkategorien und Dimensionen ist im Folgenden dargestellt:

**Kategorie**	**Subkategorie**	**Dimension**
Informationsbeschaffung	Rahmenbedingungen	Informationsüberfluss
		Ressourcen
		Akteursvielfalt
	Zugang	Person
		Internet
		Newsletter
		Fachzeitschrift
		Netzwerk
Wissenschaftliche Erkenntnisse	Nutzen	Argumentationshilfe
		Umsetzungshilfe
		Zusatzinformation
	Barrieren	Komplexität
		Statistik
		Ressourcen
		Zugänglichkeit
		Fachsprache
		Nicht vorhanden
	Einbezug in Entscheidungen	Politischer Rückhalt
		Akteurskompetenz
		Komplexität
	Strukturelle Aufbereitung	Suchmaske
		Länge
		Zusammenfassung
	Inhaltliche Aufbereitung	Übertragbarkeit
		Setting und Rahmenbedingungen
		Ergebnis
		Kosten
		Beispiele
		Statistik
		Handlungsempfehlungen
		Förderliche und hinderliche Faktoren
	Bereitstellung	Newsletter
		Veranstaltung
		Beratung
		Fachzeitschrift

Beschreibung der Datenanalyse

Die qualitative Inhaltsanalyse enthält fünf zentrale Schritte: Theoretische Vorüberlegungen, Vorbereitung der Extraktion, Extraktion, Aufbereitung und Auswertung. Im Zuge der Interviewauswertung nach Gläser und Laudel [22] wurde demnach auf Basis des theoretischen Hintergrundes ein Kategoriensystem (Suchraster) entworfen. Anhand dessen wurden Informationen, die für die Beantwortung der Forschungsfragen relevant sind, aus dem Interviewmaterial extrahiert und inhaltlich strukturiert. Die dabei entstehende Informationsbasis wurde durch die Anpassung der Kategorien an die Besonderheiten des Materials geformt. Diese Extraktion aller relevanten Daten aus den Interviews und Reflexionsbögen kann als erster Interpretationsschritt gesehen werden. Anschließend wurde das extrahierte Material aufbereitet, d. h. geprüft, nach zeitlichen oder sachlichen Aspekten sortiert und zusammengefasst. Daraufhin wurde das Material ausgewertet und analysiert.

In einem ersten Schritt wurden folglich zehn Interviews von zwei Autorinnen unabhängig auf Übereinstimmung mit den theoretisch festgelegten Kategorien untersucht und Textbestandteile dementsprechend zugeordnet. Aussagen, die nicht in das Kategoriensystem passten, wurden umformuliert und abstrahiert. Durch Selektion, Zusammenfassung und Gruppierung wurden diese Abschnitte auf die Kerninhalte reduziert, um damit weitere Subkategorien induktiv zu entwickeln. Die Vorgänge liefen aufgrund der Datenmenge simultan ab. Daraufhin erfolgte ein zweiter Durchgang der Analyse. Bei diesem wurden alle Experteninterviews anhand des erweiterten Auswertungsrasters erneut untersucht und um zusätzliche induktiv erarbeitete Kategorien ergänzt. Es erfolgten eine abschließende Überarbeitung und Überprüfung des Kategoriensystems (siehe Tabelle Z1).
